# Computational study, synthesis and evaluation of active peptides derived from Parasporin-2 and spike protein from Alphacoronavirus against colorectal cancer cells

**DOI:** 10.1042/BSR20211964

**Published:** 2021-12-08

**Authors:** Jenniffer Cruz, Miguel Orlando Suárez-Barrera, Paola Rondón-Villarreal, Andrés Olarte-Diaz, Fanny Guzmán, Lydia Visser, Nohora Juliana Rueda-Forero

**Affiliations:** 1 Universidad de Santander, Facultad de Ciencias Médicas y de la Salud, Instituto de Investigación Masira, Bucaramanga, Colombia; 2 Department of Pathology and Medical Biology, University of Groningen, University medical Center Groningen, Groningen, Netherlands; 3 Corporación Académica Ciencias Básicas Biomédicas Universidad de Antioquia, Medellín, Colombia; 4 NBC Núcleo de Biotecnología Curauma, Pontificia Universidad Católica de Valparaíso, Campus Curauma, Av. Universidad 330, Valparaíso, Chile

**Keywords:** anticancer peptides, Fmoc synthesis, hemolytic activity, intrinsic apoptosis, molecular docking, Parasporin PS2Aa1

## Abstract

Parasporin-2Aa1 (PS2Aa1) is a toxic protein of 37 KDa (30 kDa, activated form produced by proteolysis) that was shown to be cytotoxic against specific human cancer cells, although its mechanism of action has not been elucidated yet. In order to study the role of some native peptide fragments of proteins on anticancer activity, here we investigated the cytotoxic effect of peptide fragments from domain-1 of PS2Aa1 and one of the loops present in the binding region of the virus spike protein from Alphacoronavirus (HCoV-229E), the latter according to scientific reports, who showed interaction with the human APN (h-APN) receptor, evidence corroborated through computational simulations, and thus being possible active against colon cancer cells. Peptides namely P264-G274, Loop1-PS2Aa, and Loop2-PS2Aa were synthesized using the Fmoc solid-phase synthesis and characterized by mass spectrometry (MS). Additionally, one region from loop 1 of HCoV-229E, Loop1-HCoV-229E, was also synthesized and characterized. The A4W-GGN5 anticancer peptide and 5-fluorouracil (5-FU) were taken as a control in all experiments. Circular dichroism revealed an α-helix structure for the peptides derived from PS2Aa1 (P264-G274, Loop1-PS2Aa, and Loop2-PS2Aa) and β-laminar structure for the peptide derived from Alphacoronavirus spike protein Loop1-HCoV-229E. Peptides showed a hemolysis percentage of less than 20% at 100 µM concentration. Besides, peptides exhibited stronger anticancer activity against SW480 and SW620 cells after exposure for 48 h. Likewise, these compounds showed significantly lower toxicity against normal cells CHO-K1. The results suggest that native peptide fragments from Ps2Aa1 may be optimized as a novel potential cancer-therapeutic agents.

## Introduction

In the world, colorectal cancer (CRC) is one of the major causes of death. Among the most common cancers types, CRC is currently ranked fourth, after female breast, lung, and prostate cancer, with 1.93 million new cases and almost 935,000 deaths in 2020 according to the World Health Organization GLOBOCAN [1], representing about 10% of cancer cases and deaths. Overall, colorectal cancer ranks third in terms of incidence but second in terms of mortality. It is predicted that in the year 2035, the number of new cases of CRC may increase to nearly 2.5 million [2]. The annual prevalence for colon cancer in Colombia also places it as the third most frequent cancer type, after prostate and breast cancer, accounting for 8.3% of the total malignant neoplasms and with a mortality rate that locates it in the fourth place [3].

Surgery, radiotherapy, and chemotherapy are the most common treatments for colon cancer, which are very invasive and with a large number of side effects [4–8]. The development of new strategies to combat colon cancer has become urgent, and anticancer peptides (ACPs) are proposed as promising molecules. Compared with other small organic molecules and proteins, ACPs have several outstanding properties, such as small size, high activity, low immunogenicity, good biocompatibility, diversity of sequences, and multiple modification sites for the functional molecules [9]. Initially, according to their mechanism of action, ACPs were considered membrane-active peptides (necrosis) regarding their primary activity [10]. However, over the years, it was clarified that they could also be linked to different processes such as mitochondrial membrane lytic activity (apoptosis), angiogenesis inhibition, inhibition/activation of essential proteins, or recruitment of immune cells to attack cancer cells [11,12].

From a structural point of view, most ACPs have either α-helical or β-sheet conformation but some extended structures have also been reported [13]. Concerning cell targets, they can be classified into two major groups. The first one includes peptides, such as cecropins and magainins, which are active against microbial and cancer cells while being harmless to healthy mammalian cells. The second group contains ACPs, such as human neutrophil defensins HNP-1 to 3 that act against all three types of cells: microbial, normal, and cancerous [11,14].

For the design of ACPs, *in silico* techniques have been useful to save time and reduce costs in experimental tests by predicting, screening, and designing peptides with potential anticancer activity [15]. Equipments such as Support Vector Machines, and methods like molecular docking and molecular dynamics have been used in the design of ACPs [16], and also in the identification of potential anticancer molecules that inhibit important targets such as CDK2 [17], CDK5, CDK7, and CDK9 [18].

Similarly, fragmentation is a strategy to obtain short bioactive peptides from the bioactive proteins [19], e.g., HPRP-A1 exhibited a broad-spectrum anticancer activity and it is derived from the N-terminus of the ribosomal protein L1 of *Helicobacter pylori* [20]. Furthermore, Liu et al*.* [21] designed the ABH3 peptide, derived from the BH3 protein, and the data indicated that the ABH3 peptide induces cell death through the lytic properties of the peptide that disrupts cell membrane. Among others, a cyclo [EMTOVNOGQ] peptide from alpha-fetoprotein (AFP), a human protein produced during pregnancy, was tested for activity against cancer cells [22].

An interesting protein with anticancer properties is PS2Aa1, also classified as Cry46Aa1, which is produced by the Gram-positive bacterium *Bacillus thuringiensis* (*Bt*) during sporulation. This protoxin (37 kDa) is activated by serine proteases, such as proteinase K and trypsin, producing a highly toxic fragment (30 kDa) active against cancer cells [23–27]. Nevertheless, its mechanism of action or the receptors involved in its interaction with cells is still mostly unknown in detail. PS2Aa1 shares a remarkable structural similarity with Epsilon (ETX) and β-type aerolysin toxins, which are recognized as β-pore-forming toxins (β-PFTs). This similarity suggests a possible mechanism of pore action for this type of toxins [28].

Another study [24] proposed the induction of apoptosis as a mechanism of cell death together with the identification of multiple survival pathways, inhibitions including AKT, XIAP, ERK1/2, and the induction of the tumor suppressor PAR-4 after treatment with PS2Aa1. Abe et al. [29] proposed that PS2Aa1 toxins are capable of producing oligomerization, which in turn induce cell death by their binding to areas rich in membrane lipids, such as lipid rafts. It is important to note that for many of the Cry insecticidal toxins, protein receptors present in lipid rafts have been recognized, such as aminopeptidases including human aminopeptidase (h-APN) or alkaline ALP phosphatases, proteins anchored to GPI (glucosyl-phosphatidyl-inositol) [30]; hence, receptor proteins of this type may induce the activity of toxins.

Structurally, PS2Aa1, has three different domains. Domain I is rich in aromatic amino acid residues with structural diversity; it has an extended conformation of β sheets, with hydrophobic regions in the distal surface of the domain, and it is involved in the processes of insertion in the membrane and monomer oligomerization. Domain I is attributed to the primary function of recognizing membrane receptors, such as the GPI and APN anchor proteins in the glycan region. Domain II is linked to amphipathic hairpins through β chains, which are essential for pore formation. The domain III segment probably contains an amphipathic elongation consisting of a β sheet and α helix and tends to be organized similarly to aerolysins [23,28,31,32].

Therefore, the main goal of the present study was the evaluation of the anticancer activity of three natives sequences derived from PS2Aa1 domain-1, P264-G274, Loop1-PS2Aa, and Loop2-PS2Aa against colon cancer cells SW680 and SW480 using the strategy of fragmentation for the search of new bioactive compounds. These fragments were chosen based on computational analysis of the possible regions of interactions in the PS2Aa1 domain-1. Peptides sequences from the most promising regions were selected as peptides, and molecular docking simulations were performed with the APN receptor. Additionally, one fragment derived from Alphacoronavirus, Loop1-HCoV-229E, was selected because it is well-known that according to its mechanism of action, the virus interacts with the APN receptor [33]. Peptides showed to be bioactive in a dose-dependent concentration. Besides, for the most active peptide against each evaluated cancer cell line, the cellular adhesion percentage and fluorescence microscopy were evaluated to propose a possible explanation of the mechanism involved in the anticolon cancer activity.

## Materials and methods

### Materials and reagents

5-Fluorouracil (5-FU), fetal bovine serum (FBS) and sulforhodamine B (SFB) were purchased from Sigma (Sigma Aldrich, U.S.A.). Dulbecco’s modified Eagle’s medium (DMEM), L-glutamine, and penicillin/streptomycin were purchased from Lonza (Walkersville, MD, U.S.A.). Trypsin-EDTA was purchased from Invitrogen/Gibco.

### Peptide synthesis and characterization

All natives sequences from PS2Aa1 and HCoV-229E were synthesized via F-moc solid-phase peptide synthesis (SPPS) [34] using the tea-bag procedure reported by Houghten [35]. Peptides were purified by Reverse Phase-High Performance Liquid Chromatography (RP-HPLC) (Jasco Corporation, Tokyo, Japan) using a Vydac C-18 preparative column using a mixture of (A) H_2_O with 0.1% (v/v) TFA, and (B) acetonitrile (ACN) containing 0.1% (v/v) TFA as mobile phase. For the elution of peptides, the gradient program was: 30 min with 5–70% of B at 1 ml/min and detection at 220 nm. The molar mass of purified peptides was determined by ESI-MS mass spectrometry (MS) [36,37] (Supplementary Figures S1–S5).

Circular dichroism (CD) of peptides was carried out at 25°C in a 1 mm path length cuvette and spectra were obtained over 190–260 nm in a CD Spectrometer (J-815 Jasco Corporation, Japan) using a 0.2 mM peptide solution dissolved in a mixture of 50 mM sodium phosphate buffer, pH 7.4, and 30% (v/v) 2,2,2-trifluoroethanol (TFE). Each spectrum was obtained as an average of three scans taken at a rate of 20 (nm/min) with a spectral band of 1 nm. Each experiment was repeated four times and averages were taken of the resulting data [38].

### Hemolytic activity assay

The hemolytic activity of the peptides was determined by measuring the hemolysis in sheep erythrocytes induced by them, as reported in previous studies [39]. A suspension containing the erythrocytes and 0.1% Triton X-100 was used as a positive control.

The percentage of hemolysis was calculated using the following (eqn 1):

(1) $$Hemolysis\;(\%)=\frac{\left(A_{s}-A_{0}\right)}{\left(A_{100}-A_{0}\right)}\times \;100\%$$

where *A*_s_ is the absorbance of the sample, *A*_100_ is the absorbance of erythrocytes completely lysed in 0.1% Triton X-100, and *A*_0_ is the absorbance in the complete absence of hemolysis. All tests were performed independently in triplicate.

### Cell and culture conditions

All cell lines (SW480, SW620 and CHO-K1) used in this research were obtained from the Programa de Nanobiocáncer de Colombia Científica. Human epithelial colorectal adenocarcinoma cells from line SW480 and SW620 have grown in 75 cm^2^ flasks 10 ml and maintained in 10 ml of Dulbecco’s Modified Eagle Medium (DMEM) containing 10% fetal bovine serum (FBS), 2 mM glutamine, 1% MEM of non-essential amino acids, 10,000 U/ml of penicillin, 10,000 μg/ml of streptomycin and 25 μg/ml of amphotericin B. Cells were cultured at 37°C in a 5% CO_2_ atmosphere. CHO-K1, cell line derived from a biopsy of an ovary of an adult Chinese hamster, was taken as control of normal cells.

### Cytotoxicity assay

Assessment of cytotoxicity of tested peptides (P264-G274, Loop1-PS2Aa, Loop2-PS2Aa, and Loop1-HCoV-229E) and 5-FU in non-cancerous (CHO-K1) cell line and human colon cancer cells, SW480 and SW620, were performed by the SFB assay [40]. 5-FU, an established chemotherapeutic drug, and peptide A4W-GGN5, a potent antimicrobial and anticancer peptide derived from Gaegurin 5, were used as positive control. Briefly, CHO-K1, SW480, and SW620 cells at a density of 2 × 10^5^ cells/well were seeded into 96-well plates. After 24 h of incubation, the medium was removed, and cells were exposed to different concentrations of peptides and chemotherapeutic drug (from 0 to 150 μM) with the control being replaced with phosphate buffer saline (PBS). After 12 and 48 h of peptides and 5-FU treatment, without removing the medium, 50 μl of TCA (50% p/v) were added to each well, and the plates were incubated by 1 h at 4°C. After the plates were washed five times with water, they were dryed at room temperature. Then 100 μl of SRB (0.4% p/v) was added to each well and the plates were incubated for 30 min. Later on, the plates were washed five times with (1% v/v) acetic acid and dried at room temperature, and the colorant SRB was dissolved in 200 μL of buffer Tris 10 mM (pH 10.5). Finally, after gently mixing, the absorbance of each well was evaluated at 450 nm in a Varioskan™ LUX multimode microplate reader (Thermo Fisher Scientific, Waltham, MA, U.S.A.). Experiments were done in triplicate and results are presented as the percentage of inhibition of non-treated CHO-K1 or SW480 and SW620 cells. Data represent the mean and standard deviation of three independent experiments (*n*=3). The concentration at which the chemotherapeutic agent and the peptides effectively reduced cell viability by 50% (EC_50_) for each cell line, was calculated in GraphPad Prism 4.03 software (GraphPad Software, Inc., La Jolla, CA, U.S.A.) using non-linear regression.

### *In vitro* binding assay

SW480 and SW620 cells at a density of 2 × 10^5^ cells/well were seeded into 24-well plates. After 24 h of incubation, cell binding was examined by inoculating the suspension with 750 μl of the rhodamine-labeled peptide at different concentrations (from 0 to 150 μM). After 1 h of incubation at 37°C and 5% CO_2_ atmosphere, a constant volume of the supernatant (containing non-adherent peptides) was collected from the well, and the fluorescence of the supernatant was measured in a Varioskan™ LUX multimode microplate reader (Thermo Fisher Scientific, Waltham, MA, U.S.A.) (λex = 546 nm; λem = 568 nm). The assay was performed using six replicates for each peptide in SW620 and SW480 cells from the same number of passes. The adherent fraction of the peptide was measured by interpolation with a calibration curve for each peptide. The test results were expressed as percent binding, taking into account the relationship between the initial concentration of the peptide and the concentration of adherent peptide [41].

### Fluorescence microscopy

The assays for determining the localization of the peptides in the SW620 and SW480 cells were carried out as previously published [42]. In detail, the cells were seeded into chamber slides (2 × 10^5^ cells/well) for 24 h, washed twice with PBS, and then incubated in the absence and presence of the most active rhodamine-labeled peptides for each of cell line, at a concentration twice the EC_50_ for 1 h at 37°C in the dark. After that, the monolayers were washed three times with PBS (1 mM, pH 7.2) to remove the excess peptide, and the cells were fixed with a 4% (v/v) solution of p-formaldehyde in PBS for 30 min. The cell monolayers were washed with PBS and the core was stained with 1 mg/ml of Hoechst for 2 min at room temperature. The monolayers were analyzed in a Nikon Eclipse TE2000-E fluorescence microscopy.

### Annexin V-Cy3 staining

The presence of phosphatidylserine at the cell surface was detected by phosphatidylserine binding protein annexin V conjugated with Cy3 using the commercially available Annexin V-Cy3 apoptosis detection kit (APOAC, Apoptosis Detection Kit, Sigma). The cells (2.0 × 10^5^ ml^−1^) were seeded into chamber slides for 24 h, washed twice with PBS, and then incubated in the absence and presence of the most active peptides with the respective EC_50_ concentrations of the most active peptides for each of cell line (SW480 and SW620) for 24 and 48 h. The PBS and binding buffer were used to wash the adherent cells and the washed cell adherents were suspended in 50 µl of Ann-Cy3 and 6-CFDA. Then, the plates were incubated for 10 min in the dark. Afterwards, the excess label was removed by washing the cells with binding buffer. EVOS M7000 digital inverted fluorescence microscope (ThermoFisher Scientific; Waltham, MA, U.S.A.) was used to observe Ann-Cy3 and 6-CFDA-labeled cells. This assay allowed one to differentiate live cells (green), necrotic cells (red), and apoptotic cells (red nuclei and green cytoplasm). The percentage of cells reflecting apoptotic and necrotic cells was manually calculated. Data were collected for three replicates and used to calculate the respective means and standard deviations [24].

### Caspase 3-7 assay

To measure the specific activity of caspase 3 and 7, a assay kit named CellEvent™ Caspase-3/7 Green Detection Reagent (Invitrogen™) was used. This assay provides a proluminescent caspase-3/7 substrate containing a sequence (DEVD) specific to caspase 3 and 7. If caspases 3 and/or 7 are active, the substrate is cleaved and aminoluciferin will be emitted. Briefly, chamber slides were seeded with 50 μl of cancer cells (for SW480 and SW620, 20000 cells/well) resuspended in the culture medium. Plates were incubated at 37°C, 5% CO_2_ for 24 h. Cells were loaded with 7.5 μM CellEvent™ Caspase-3/7 Green Detection Reagent then treated at EC_50_ concentration of more active peptide for each cell and incubated for another 4 h. After that, the monolayers were washed three times with PBS (1 mM, pH 7.2) to remove the excess peptide, and the cells were fixed with a 4% (v/v) solution of p-formaldehyde in PBS for 30 min. About 10 µL of Entellant were added each sample and mounted with a glass coverslip. The monolayers were analyzed in a Nikon Eclipse TE2000-E fluorescence microscopy [43].

### LDH assay

Lactate dehydrogenase (LDH) is a cytosolic enzyme present in many different cell types that is released into the cell culture medium upon damage to the plasma membrane. The CyQUANT LDH Cytotoxicity Assay was used to accurately and quantitatively measure this extracellular LDH. For this test 10,000 cells/100 µl (the optimal number) were plate in triplicate wells in a 96-well tissue culture plate with additional wells for controls (Spontaneous LDH Activity and Maximum LDH Activity). Cells were incubated overnight at 37°C with the appropriate level of CO_2_. After overnight incubation, samples were preaparated according to the following: was added 10 μl of sterile, ultrapure water to one set of triplicate wells of cells, was added nothing to one set of triplicate wells of cells and was added 10 μl of the peptides to one set of triplicate wells of cells. To the set of triplicate wells serving as the Maximum LDH Activity Controls, was added 10 µl of 10× Lysis Buffer, then mix by gentle tapping. The plate was incubated at 37°C with the appropriate level of CO_2_ for 45 min. Then, 50 µl of the supernatant were transferred of each sample (Spontaneous LDH Activity, Maximum LDH Activity, and Chemical-treated LDH activity) to a 96-well flat-bottom plate in triplicate wells. Aliquot of 50 µl of reaction mixture was placed to each sample well, then mixed well. The plate was incubated at room temperature for 30 min protected from light. About 50 µl of Stop Solution was added to each sample, then the absorbance at 490 and 680 nm was measured [44].

### Molecular docking of peptides and APN receptor

The 3D structure of the peptides was predicted using the software PEP-FOLD 3.5 [45], and the 3D structure of the APN receptor was obtained from the Protein Data Bank [46] (PDB ID: 6ATK), keeping only the A chain.

For the two peptides that produced the best experimental results, two rounds of molecular docking simulations were performed to predict their binding with the APN receptor by using the software tool Rosetta (version 3.12) [47]. In the first round, 500 models were obtained by using a global simulation, i.e., the peptide could move around the APN receptor. For this round, the flags used in Rosetta were set as follows: -nstruct 500, -dock_pert 3 3, -spin, -randomize1, -randomize2, -ex1 and -ex2aro. Later on, the number of possible hydrogen bonds between the peptide and the APN was obtained for each of the 500 models, and the most frequent residues involved in hydrogen bond interactions were identified.

Additionally, for each peptide, the model with the highest number of hydrogen bonds, and the peptide located near to the most frequent residues, were selected to be used in the refinement step by using the FlexPepDock protocol of Rosetta with the following flags: -nstruct 100, -flexPepDocking:flexpep_score_only, -flexPepDocking:pep_refine, and the Rosetta FlexPepDock web server [48] (version 3.2) was also used with the number of low and high-resolution structures equal to 100. Finally, the resulting models were analyzed in terms of hydrogen bonds with the APN receptor.

### Statistical analysis

All experiments were repeated at least three times. Results were expressed as mean values ± standard error (mean ± SE). Significant differences between the treatments and their respective controls were determined based on one-way ANOVA followed by Tukey’s test. A level of *P*<0.05 was considered to be significant.

## Results

### Design and characterization of the peptides

Based on previous journal publications [49] and results obtained by a computational analysis in our research group, it was possible to identify some probable regions of PS2Aa1 that can interact with the h-APN receptor. Based on these considerations, the PS2Aa1 was fragmented, and three peptides, namely P264-G274, Loop1-PS2Aa and Loop2-PS2Aa, were derived from this toxin, and their synthetic forms were synthesized by Fmoc methodology. Additionally, one region from loop 1 of HCoV-229E, Loop1-HCoV-229E, was synthesized to use as a control, since it has been shown that the HCoV-229E acts through its interaction with the h-APN receptor [33].

Here, the biological activity of peptides derived from different loops in the PS2Aa1 and HCoV-229E was evaluated against colon cancer cells. Peptides presented different physicochemical properties, as shown in Table 1. The net positive charge of peptides varies from +1 to +3. According to Heliquest [50], P264-G274 peptide has 63.64% of polar residues and 36.36% of non-polar residues, a hydrophobicity (*H*) value of 0.19, and a hydrophobic moment (μH) value of 0.30; besides, it is an uncharged peptide. Loop1-PS2Aa and Loop2-PS2Aa peptides presented *H* values of 0.17 and 0.56, respectively. μH values of Loop1-PS2Aa and Loop2-PS2Aa peptides were 0.23 and 0.34, respectively. Loop1-PS2Aa peptide presented a net positive charge of +1, while Loop2-PS2Aa peptide presented a net positive charge of +2.

**Table 1:** Peptides sequence, molecular characterization and physicochemical properties

Peptide	Sequence	Number of residues	*z*	Molecular weight (Da)		*H*	μH
				Theoretical	Observed		
P264-G274	PARDVLNTTSG-NH_2_	11	+1	1129.29	1130.23	0.19	0.38
Loop1-PS2Aa	NNETYFNAVKP-NH_2_	11	+1	1295.47	1295.49	0.17	0.23
Loop2-PS2Aa	TYFNAVKPPITA-NH_2_	12	+2	1320.61	1320.63	0.56	0.34
Loop1-HCoV-229E	FKPQSGGGKCF-NH_2_	11	+3	1154.41	1154.44	0.33	0.35
A4W-GGN5	FLGWLFKVASK-NH_2_	11	+3	1294.67	1294.68	0.80	0.50

*z* (net positive charge) (https://www.bachem.com/service-support/peptide-calculator/), *H* (hydrophobicity), μH (hydrophobic moment).

Loop1-HCoV-229E, a unique peptide derived from HCoV-229E spike protein, presented an *H* value of 0.33 and a μH value of 0.35. Additionally, this was the peptide with the highest net positive charge (+2) along with the control peptide A4W-GGN5. Peptides were characterized by MS, and all the peptides presented the same theoretical and experimental mass (*m/z*), as shown in Table 1.

**Figure 1:**
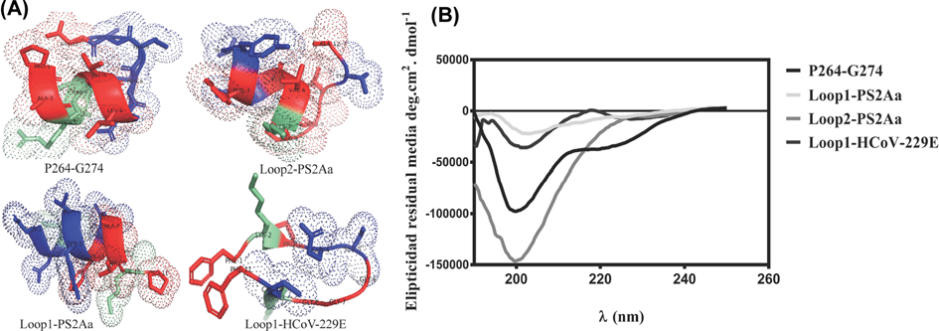
Secondary structure of peptides (**A**) Theoretical *in silico* secondary structure of P264-G274, Loop1-PS2Aa, Loop2-PS2Aa, and Loop1-HCoV-229E. (**B**) Circular dichroism spectra of the peptides in TFE (30% p/V). CD was recorded after four accumulations at 20°C, using a 1 mm path length quartz cell, between 190 and 250 nm at 100 nm min^−1^, with a bandwidth of 0.5 nm. Peptide concentration: 1 mg/ml.

On the other hand, the theoretical *in silico* secondary structures of the peptides obtained with the software PEP-FOLD 3.5 [45] were compared with the CD spectra. All the peptides exhibited a theoretical α-helix secondary structure (see Figure 1), except for the Loop1-HCoV-229E peptide that exhibited a theoretical β-lamellar secondary structure. The secondary structure of the peptides was also confirmed by the determination of their CD spectra. Figure 1 shows the CD spectra of all the synthesized compounds, in which all PS2Aa1 derivatives exhibited a maximum absorption band at 190 nm and two minimum absorption bands between 205 and 220 nm (see Figure 1A,B). On the other hand, the CD spectra for alphacoronavirus-derived peptides exhibited a secondary structure of β-sheets, with a maximum absorption band at 195 nm and a minimum absorption band at 216 nm.

### Anticancer and hemolytic activities

The anti-proliferative response of the peptides and the chemotherapeutic drug (5-FU) on SW480 and SW620 were examined using the SRB assay. The anticancer activities P264-G274, Loop1-PS2Aa, Loop2-PS2Aa, and Loop1-HCoV-229E peptides against two different cancer colon cell lines are shown in Table 2. For all cell lines tested, Loop1-PS2Aa exhibited a stronger anticancer activity against SW480 after exposure for 48 h, whereas the most active peptide against SW620 was P264-G274 at the same exposure time.

**Table 2:** EC_50_ and HC_50_ values of the anticancer and hemolytic activity of the peptides

Peptide	EC_50_ (μM)			%HC_50_ at 150 μM
	SW480	SW620	CHO-K1	
P264-G274	90.98 ± 0.75	11.28 ± 0.52	—	12.2%
Loop1-PS2Aa	23.76 ± 1.25	106.2 ± 1.67	----	18.1%
Loop2-PS2Aa	92.99 ± 0.98	15.95 ± 0.69	----	9.4%
Loop1-HCoV-229E	125.0 ± 1.32	>150.0	----	9.2%
A4W-GGN5	98.63 ± 1.17	22.07 ± 1.63	----	23.0%
5-FU	24.38 ± 0.82	11.92 ± 1.20	----	------

— Undetermined.

**Figure 2:**
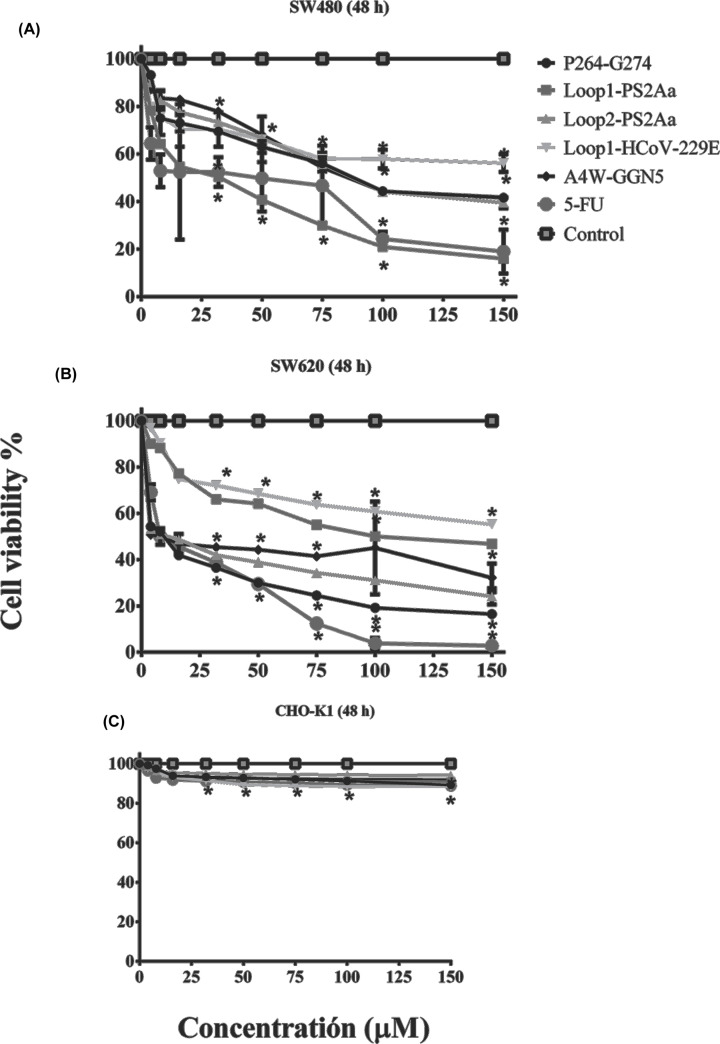
Effect of peptides (P264-G274, Loop1-PS2Aa, Loop2-PS2Aa, Loop1-HCoV-229E, and A4W-GGN5) and the chemotherapeutic drug (5-FU), on the viability of -cancerous and non-cancerous cells measured by the SRB assay A4W-GGN5 and 5-FU were used as a positive control. (**A–C**) SW480, SW620, and CHO-K1 cells were incubated with peptides in the concentration range of 0–150 μM for 48 h. Dosages that caused a statically significant decrease in cell growth compared with the untreated control at each time point were indicated by asterisks (*P< 0.05; one-way ANOVA followed by Tukey’s test).

The results show dose-dependent inhibition of cell growth at 48 h, as illustrated in Figure 2A–C. The ability of all peptides to inhibit the growth of SW480 and SW620 cells is significant from the concentration of 4 μM on. Within all cell lines tested, Loop1-PS2Aa exhibited stronger anticancer activity against SW480 after exposure for 48 h, whereas the most active peptide against SW620 was P264-G274 at the same time of exposure. At 48 h of exposure, the peptides did not exhibit significant inhibition of normal CHO-K1 cells (Figure 2C). Furthermore, the peptides were more active against SW620 cells than against SW480 cells. 5-FU exhibited significant inhibition of the growth from 48 h of treatment in both cell lines.

The percentages of viability SW480 cells at the highest peptides concentration (150 μM) were as follows: Loop1-PS2Aa peptide 16%; Loop2-PS2Aa peptide 39.5%; peptide P264-G274 41.6%; Loop1-HCoV-229E peptide 56.2%. In this case, the Loop1-PS2Aa peptide was more active than the positive controls 5-FU (18.9% viability) and A4W-GGN5 (41.8% viability) (Figure 2A). In the case of SW620 cells, the percentages of cell viability at the same peptide concentration were: P264-G274 16.5%; Loop2-PS2Aa peptide 24.1%; Loop1-PS2Aa peptide 46.8%; Loop1-HCoV-229E peptide 55.2%. 5-FU exhibited strong activity on SW480 cells with a viability percentage of 2.8%, while the value for the A4W-GGN5 peptide was of 32.2% (Figure 2B).

The anticancer activities of P264-G274, Loop1-PS2Aa, Loop2-PS2Aa, and Loop1-HCoV-229E peptides against two different colon cancer cell lines are shown in Table 2. The peptides presented anticancer activity with EC_50_ values ranging from 11.28 μm to more than 150 μM. Loop1-PS2Aa peptide showed higher activity (EC_50_ 23.76 μM) than 5-FU (EC_50_ 24.38 μM)- against SW480, while the EC_50_ values of P264-G274, Loop2-PS2Aa, Loop1-HCoV-229E, and A4W-GGN5 peptides were 90.98 μM, 92.99 μM, 125.0 μM, and 98.63 μM, respectively. Moreover, the peptide P264-G274 showed the highest EC_50_ value of 11.28 μM, followed by 5-FU (11.92 μM), Loop2-PS2Aa (15.95 μM), A4W-GGN5 (22.07 μM) Loop1-PS2Aa (106.2 μM) and Loop1-HCoV-229E peptides (>150.0 μM). The peptides were more active against the SW620 cells than against SW480 cells after 48h of exposure.

**Figure 3:**
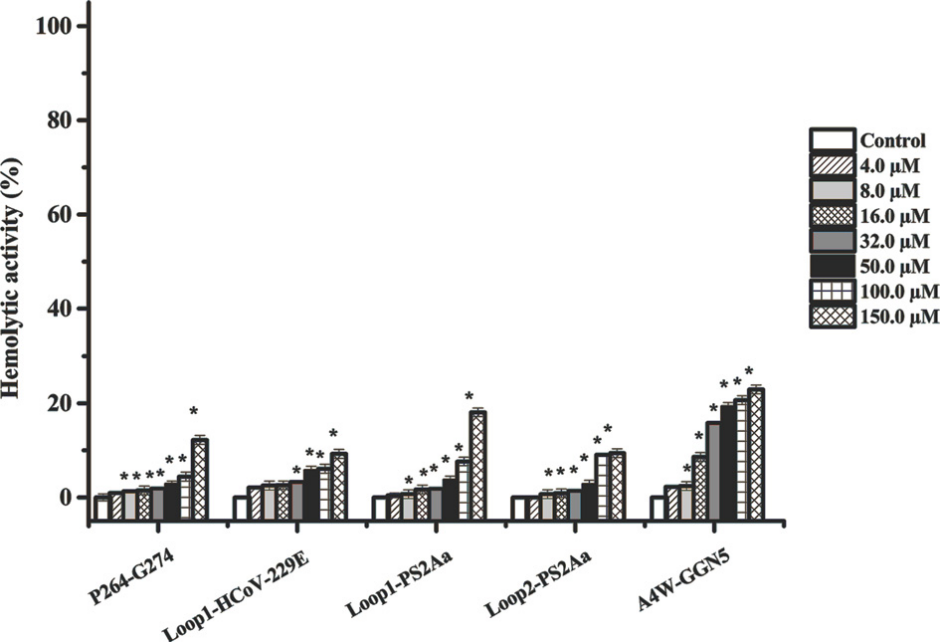
Hemolytic activity of peptides (P264-G274, Loop1-PS2Aa, Loop2-PS2Aa, Loop1-HCoV-229E, and A4W-GGN5 Hemolytic activity of peptides against human red blood cells at different peptide concentrations (4–100 μM), in Hank’s glucose at 37°C for 4 h of exposure. Experiments were performed in three independent replicates. Dosages that caused a statically significant decrease in cell growth compared with the untreated control at each time point were indicated by asterisks (*P<0.05; one-way ANOVA followed by Tukey’s test).

The degree of hemolysis of the peptides derived from PS2Aa1 was determined on human erythrocytes. The results presented in Figure 3 and Table 2 show that all evaluated peptides exhibited an HC_50_ value of less than 150 μM, with a percentage of hemolysis less 24% lower than the positive control (TX-100 0.1%). These results indicate that all compounds evaluated may be promising candidates as anticancer agents, without producing a negative effect on red blood cells.

### *In vitro* binding assay

**Figure 4:**
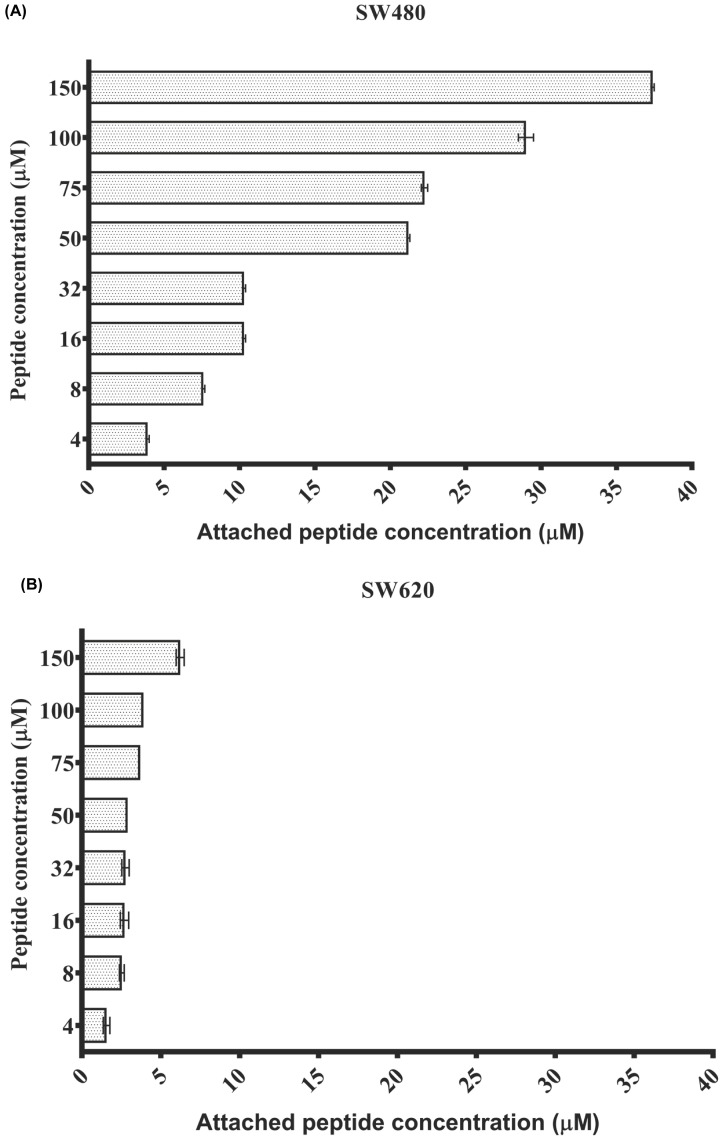
*In vitro* cell binding of the most active peptides (**A**) Cell binding of Loop1-PS2Aa peptide on the cancerous cell line SW480. (**B**) Cell binding of P264-G274 peptide on the cancerous cell line SW620. Experiments were performed in three independent replicates.

*In vitro* cell binding of the most active peptides was evaluated at an 1 h incubation period with the compounds labeled with rhodamine fluorochrome. The concentration of the attached peptide was calculated indirectly by quantifying the fluorescence of each of the supernatants of the peptides at different concentrations and by performing the calibration curve of each of the most active peptides against the SW480 cell lines and SW620. The results show that at low peptide concentrations, the percentages of cell binding are high, but as the concentration increases, they decrease probably when approaching the saturation level of the molecules in contact with the cell membrane (Figure 4).

In Figure 4, the Loop1-Ps2Aa peptide, the most active against the SW480 cell line, showed that at its highest concentration (150 μM) 19.3% of it has bound (37.4 μM). In contrast, at low concentrations, 4, 8, and 16 μM, the percentages of binding were 100%, 95.3%, and 63.5%, respectively.

On the other hand, the P264-G274 peptide presented a very low level of cell binding at all the concentrations evaluated with SW620 cells. At 4 μM concentration, only 35.3% of the cells binding, while at the highest peptide concentration (150 μM) only 4% had adhered or penetrated the cell. These results show a greater difficulty of adhesion or entry of the P264-G274 peptide on the SW620 cells when compared with the binding of the Loops1-PS2Aa peptide to the SW480 cells. The number of residues with which the peptide chain of P264-G274 can interact with the receptors present in SW620 cells may be less.

### Fluorescence morphological evaluation

**Figure 5:**
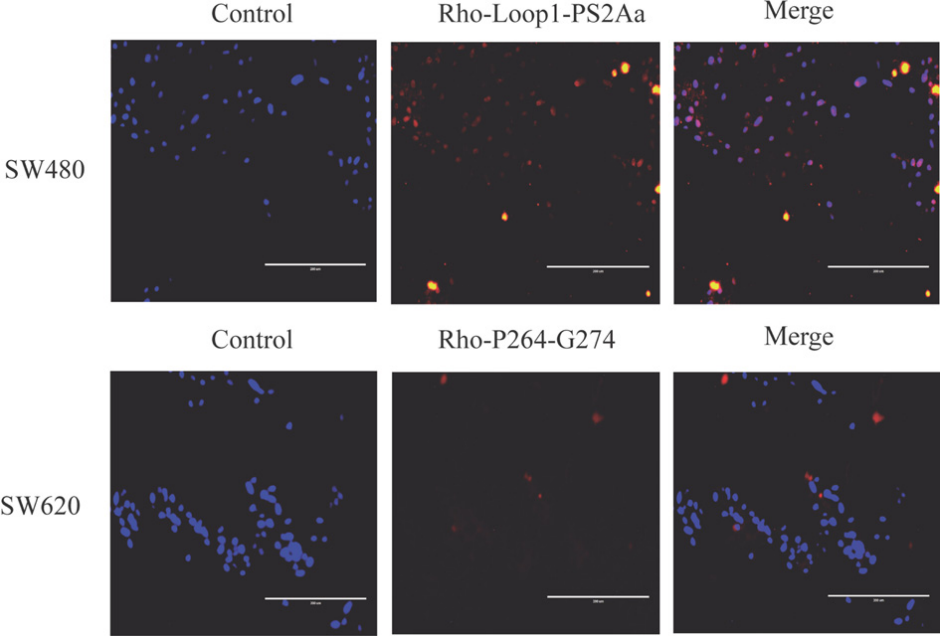
SW480 and SW620 colon cancer cells were stained by Hoechst and observed under fluorescence microscopy (20×) Cell morphology was observed under fluorescence microscopy.

The Hoechst double staining of SW480 and SW620 cells is represented in Figure 5. Under fluorescence microscopy, more uniformly blue live cells with normal morphology were observed in the control of untreated cells than in the peptides treated cells. The colocalization of the peptide Rho-Loop1-PS2Aa at 47.5 μM resulted in the entering of compounds into the nucleus of SW480 cells, while the peptide Rho-P24-G274 was located in cytoplasmic regions, without affecting the nucleus. These results were observed after 1 h of exposure. These findings suggest that Rho-Loop1-PS2Aa and Rho-P24-G274 can be able to interact with nuclear and cytoplasmic proteins on SW480 and SW620 colon cancer cells.

### Annexin V-CY3 staining

**Figure 6:**
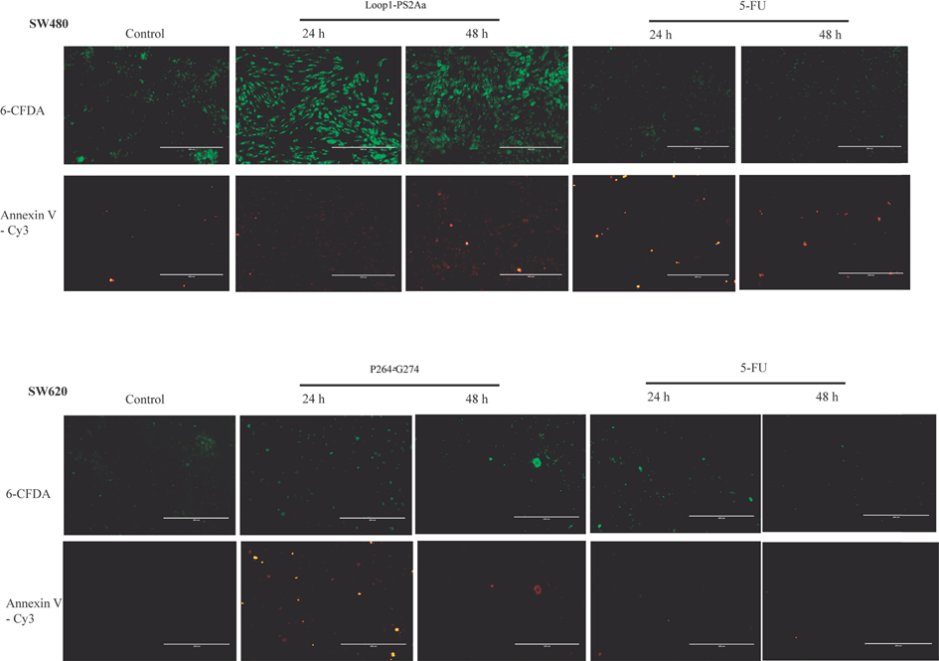
Photomicrographs of control and Annexin V-Cy3/6-CFDA stained SW480 and SW620 colon cancer cells treated with the peptides Loop1-PS2Aa and P264-G274, respectively for 24 and 48 h. 5-FU was taken as a positive control

The early apoptotic cells are differentiated using the combination of annexin V-Cy3 (red emission) and 6-CFDA (green emission). In the case of early apoptosis, both annexin V-Cy3 and 6-CFDA are positive (green and red emission), whereas in necrosis only annexin V-Cy3 is positive (only red emission) and in viable cells, only 6-CFDA is positive (only green emission). The results are given in Figure 6. When the cells were treated with most active peptides for each of the cell lines, Loop1-PS2Aa and P264-G274, both annexin V-Cy3 and 6-CFDA are positive which clearly indicates the early stage of apoptosis. Same results were observed with the control of chemotherapeutic drug 5-FU.

### Caspase and LDH mechanism

**Figure 7:**
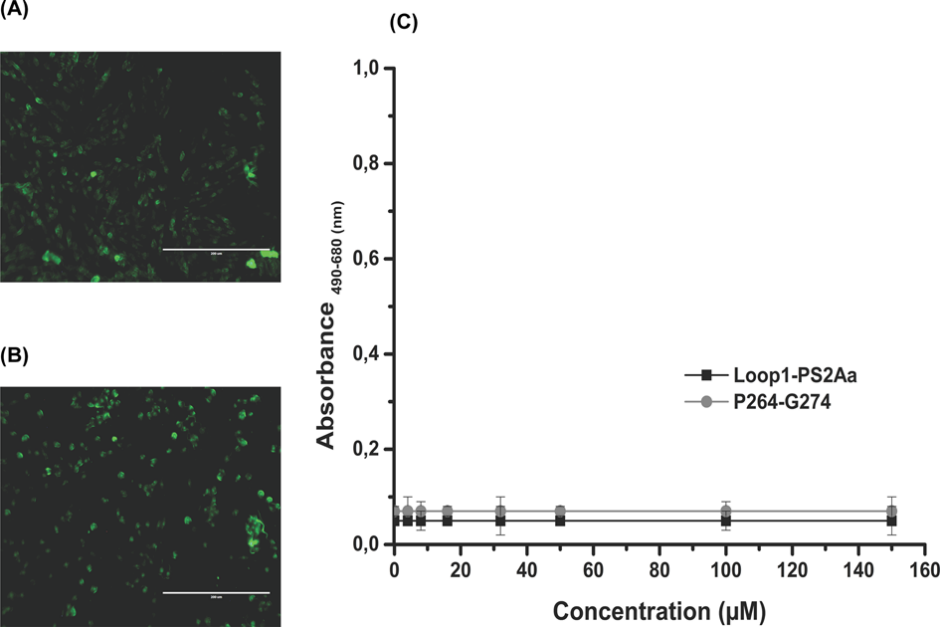
Caspase and LDH assay (**A**) intrinsic apoptosis of Loop1-PS2Aa peptide on the cancerous cell line SW480 after 4 h of exposure. (**B**) intrinsic apoptosis of P264-G274 peptide on the cancerous cell line SW620 after 4 h of exposure. (**C**) No detection of LDH enzyme in the extracellular medium after 4 h of exposure with the peptides Loop1-PS2Aa peptide on the cancerous cell line SW480 and P264-G274 peptide on the cancerous cell line SW620. Experiments were performed in three independent replicates.

Recent data suggest that caspase-3 and caspase-7 must have distinct functions. Caspase-3 can inhibit ROS production and is the effector caspase necessary for efficient cell killing. In contrast, caspase-7 has no significant role in sensitivity to intrinsic cell death, but it is responsible for ROS production and cell detachment. The action mechanism of Peptides Loop1-PS2Aa and P264-G274 against SW480 and SW620, respectively, is mediated by intrinsic apoptosis (green cells Figure 7A,B). Likewise, for the effect of the peptides over SW480 and SW620 in the extracellular medium no LDH enzyme was detected (Figure 7C).

### Molecular docking of peptides and h-APN receptor

**Figure 8:**
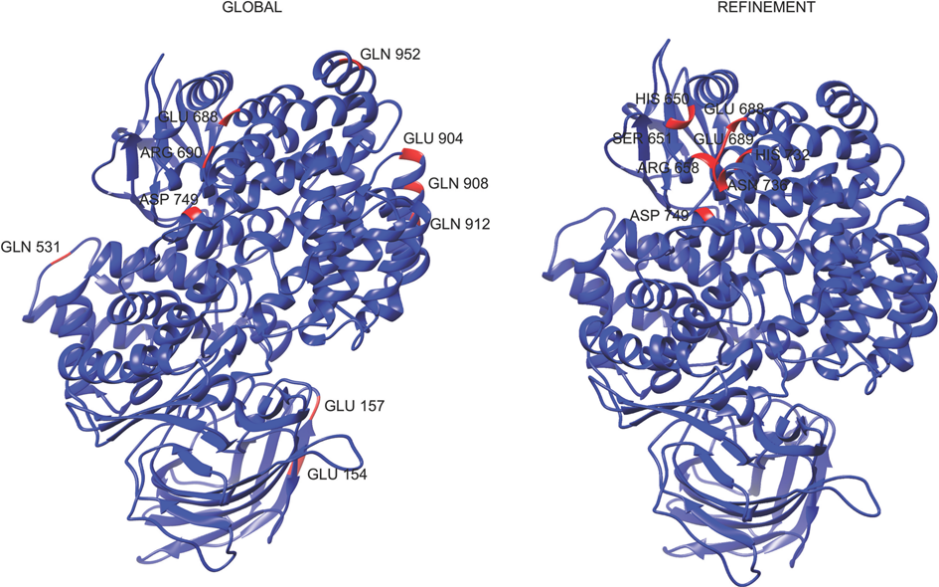
APN residues involved in hydrogen bonds with P264-G274 peptide This figure shows the top 10 of the APN residues involved in hydrogen bonds with peptide P264-G274, in 500 models obtained in global simulations (left) and 100 models obtained in the refinement round (right).

**Figure 9:**
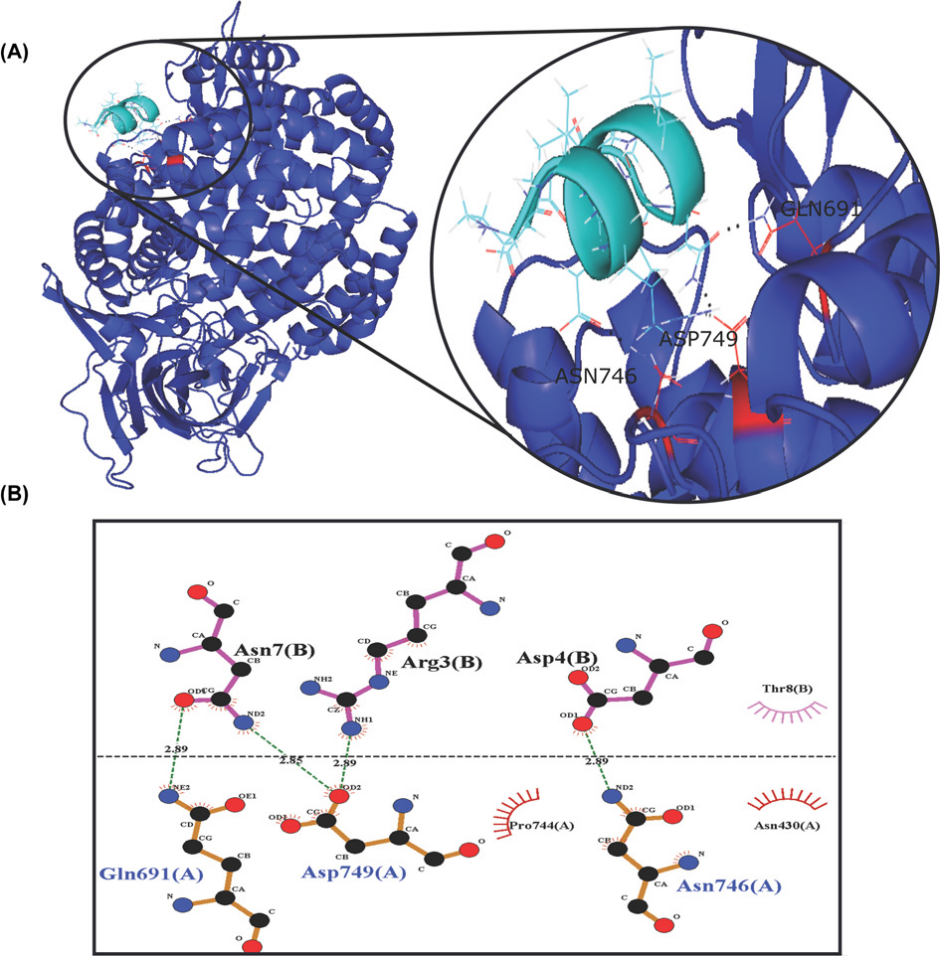
Interactions in the resulting complex between P264-G274 peptide and APN (**A**) Complex between the P264-G274 peptide and APN. (**B**) Hydrogen bonds and hydrophobic interactions found in the complex.

The computational results obtained suggest that peptide P264-G274 has a preference for interacting with amino acid residues such as Gln and Glu when it is free to move around the h-APN monomer. In the refinement step, where this peptide could only move in a specific region of the APN, the preferred residues were Asp^749^, Gln^691^, Arg^690^, and Glu^689^. Table 3 shows the top 10 residues in the APN with their corresponding number of hydrogen bonds found in the 500 models obtained in the global simulations, and the top 10 of APN residues found in the 100 models predicted in the refinement step. Moreover, Figure 8 shows the top 10 residues in the 3D structure of the APN for both rounds of simulation, and Figure 9 shows the interactions of the resulting complex between the peptide P264-G274 and the APN.

**Table 3:** Top 10 of the APN residues that were found to interact with peptide P264-G274 through hydrogen bonds

Residue	Number of hydrogen bonds in 500 models	Residue	Number of hydrogen bonds in 100 refined models
Arg^690^	15	Asp^749^	126
Gln^908^	12	Gln^691^	38
Gln^912^	11	Arg^690^	31
Asp^749^	10	Glu^689^	20
Glu^157^	9	Arg^658^	3
Glu^904^	7	Glu^688^	3
Glu^688^	7	His^650^	3
Gln^531^	7	His^732^	2
Gln^952^	**7**	Ser^651^	1
Glu^154^	7	Asn^736^	1

**Figure 10:**
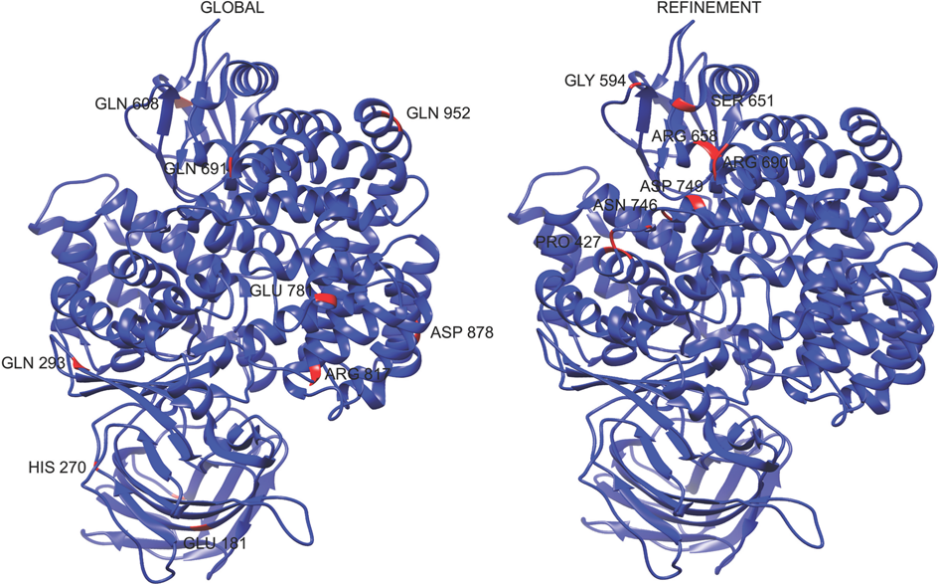
APN residues involved in hydrogen bonds with Loop1-PS2Aa peptide This figure shows the top 10 of APN residues involved in hydrogen bonds with peptide P264-G274, in 500 models obtained in global simulations (left) and 100 models obtained in the refinement round (right).

**Figure 11:**
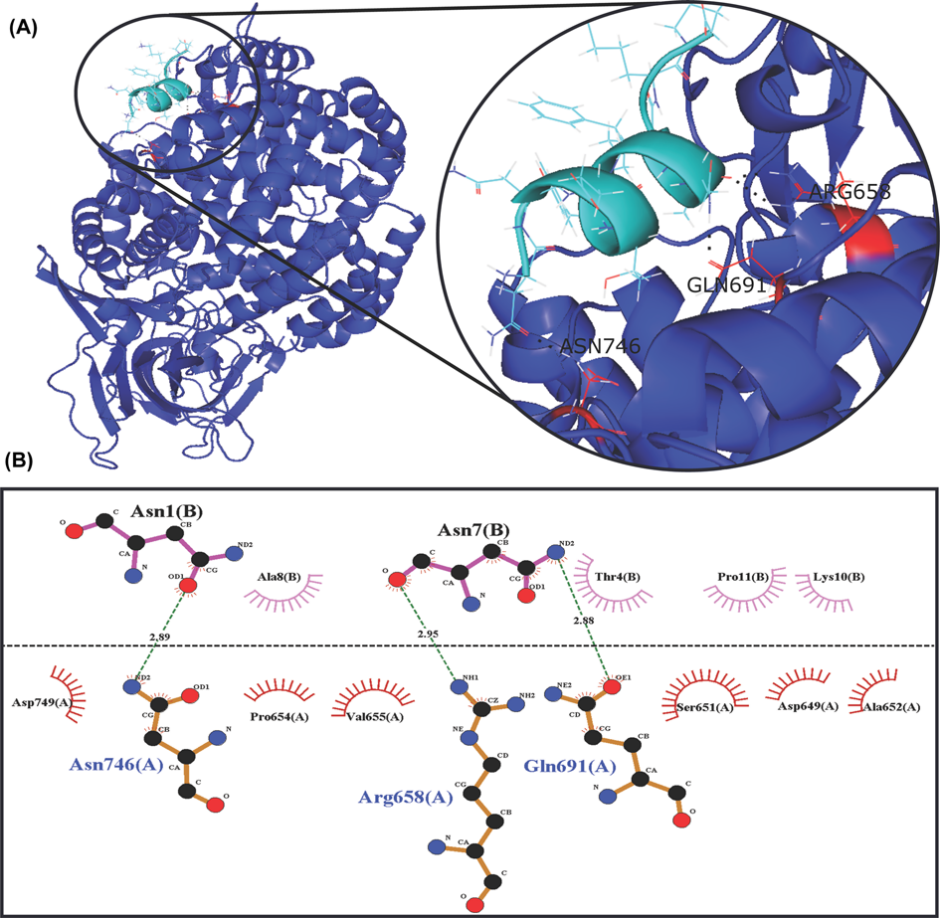
Interactions in the resulting complex between Loop1-PS2Aa peptide and the APN (**A**) Complex between the Loop1-PS2Aa peptide and APN. (**B**) Hydrogen bonds and hydrophobic interactions found in the complex.

Similarly, the peptide Loop1-PS2Aa peptide shows a preference to interact with residues such as Asp, Gln, and Glu in global simulations, and a preference for Asn^746^, Arg^658^, and Asp^423^ in the refinement round. Table 4 shows the top 10 of the APN residues involved in hydrogen bonds with this peptide in both, global and refinement simulations. Moreover, Figure 10 shows these top residues highlighted in red color, and Figure 11 shows the interactions of the resulting complex between the peptide Loop1-PS2Aa and APN.

**Table 4:** Top 10 of the APN residues that were found to interact with peptide Loop1-PS2Aa through hydrogen bonds

Residue	Number of hydrogen bonds in 500 models	Residue	Number of hydrogen bonds in 100 refined models
Asp^878^	7	Asn^746^	165
Gln^691^	7	Arg^658^	162
Glu^781^	6	Asp^423^	14
Asp^118^	5	Ser^651^	6
Arg^817^	5	Gln^691^	4
Gln^293^	5	Asn^430^	1
Gln^952^	5	Pro^427^	1
Gln^608^	5	Gly^594^	1
His^270^	5	Arg^690^	1
Glu^181^	5	Asp^749^	1

Finally, Table 5 summarizes of the APN and peptides residues involved in the hydrophobic and hydrogen bond interactions for the best complexes found for P264-G274 and Loop1-PS2Aa peptides.

**Table 5:** Summary of APN and peptides residues involved in molecular interactions in the best complexes

Peptide	Hydrogen bonds^*^	Hydrophobic interactions^*^
P264-G274	Arg3(B) - Asp749(A)	A: Asn430, Pro744
	Asp4(B) - Asn746(A)	B: Thr8
	Asn7(B) - Asp749(A)	Asn7(B) - Gln691(A)
Loop1-PS2Aa	Asn1(B) - Asn746(A)	A: Asp649, Ser651, Ala652, Pro654, Val655, Asp749 B: Thr4, Ala8, Lys10, Pro11
	Asn7(B) - Arg658(A)	
	Tyr5(B) - Gln691(A)	

* A = APN, B = Peptide.

## Discussion

Current cancer treatment is often accompanied by several detrimental side effects; for instance, toxic effects of 5-FU, a chemotherapeutic drug that is usually used for metastatic colon cancer treatment [51]. Besides, the development of drug resistance by cancer cells has limited the success of chemotherapeutic drugs. Therefore, a safe and selective anticancer agent with a new mode of action has been evaluated. Parasporin-2Aa1 (PS2Aa1) was shown to be cytotoxic against specific human cancer cells. In order to study the role of some native peptide fragments of proteins on anticancer activity, here we investigated the cytotoxic effect of peptide fragments from domain-1 of PS2Aa1 and one of the loops present in the binding region of the virus spike protein from Alphacoronavirus (HCoV-229E). In this respect, P264-G274 (PARDVLNTTSG**-**NH_2_) and Loop1-PS2Aa (NNETYFNAVKP-NH_2_)- amphipathic cationic ACPs, could be a choice. The reported results demonstrate that P264-G274 and Loop1-PS2Aa peptides exhibit time and concentration-dependent manner of growth inhibition of SW620 and SW480 cells, respectively. These may be due to the electrostatic interaction of positive charges (from +1 to +3) in the peptides with the negative charge of anionic molecules, such as phosphatidylserines, and glycosaminoglycans, which make the cancer cell membranes similar in composition to the bacterial membranes [52]. The initial electrostatic interactions between the cationic peptide with the negatively charged cancer cell membranes are important to destabilize the membrane [53]. Subsequently, peptides may rapidly penetrate the cancer cells with a hydrophobic property (36.36% of nonpolar residues in P264-G274 and 45.45% in Loop1-PS2Aa peptides). The other peptides, like Loop2-PS2Aa and Loop1-HCoV-229E, were not significantly inhibited despite having a higher net positive charge than the more active peptides (Table 1). So also, the presence of some residues such as Trp becomes essential in the sequence. Therefore, our results suggest that a combination of P264-G274 and Loop1-PS2Aa peptides with 5-FU can exert a synergistic effect and enhance the efficacy of therapeutics.

Peptides structure plays a central role in their activity. It is commonly accepted that most ACPs do not fold in a well-defined structure when free in solution but adopt α-helix or β-sheet structure when electrostatic interactions with membranes occur [53]. Differences in terms of structure were the first method for the classification of ACPs. Examples of some AMPs, lately defined as α-ACPs, are cecropin, magainin, melittin, and buforin II, with lactoferricin B, HNP-1/3, and gamesin being classified as β-ACPs W [54]. More recently, it was noticed that, independently of the secondary structure that the peptide adopts, a classification considering the mechanisms of action in the target cancer cells was more suitable [54]. Initially, ACPs were related to necrosis processes as their primary activity. However, over the years, it was clarified that they could also target different processes, such as mitochondrial membrane lytic activity (apoptosis), targeting essential cell proteins, inhibiting angiogenesis or recruiting immune cells to attack cancer cells and non-membrane activities [54]. These ACPs have high selectivity toward cancer cell membranes and develop low resistance compared to conventional chemotherapeutic drugs. From a structural point of view, P264-G274 (EC_50_ = 11.28 against SW620) and Loop1-PS2Aa (EC_50_ = 23.76 against SW480) peptides with the alpha-helix secondary structure were found to be more active compared with the peptide derived from alpha-coronavirus Loop1-HCoV-229E (EC_50_ = 125.0 μM and higher than 150.0 μM against SW480 and SW620, respectively). Furthermore, these compounds presented a lower EC_50_ value compared with the 5-FU positive control and the A4W-GGN5 peptide for each of the cell lines where they were most active (Table 2).

The present study is the first one carrying out the fragmentation of the toxic protein PS2Aa to understand the role of some regions of domain I, knowing in advance that the protein has exhibited high anticancer activity [17]. This domain is composed of four short alpha-helices and beta antiparallel sheets, which is also characterized by being rich in exposed aromatic amino acid residues (Phe, Trp, and Tyr), suggesting a possible site of binding of PS2 to susceptible cancer cells. Recently, the role of GPI-anchored proteins in the mechanism of action of PS2 toxins has been studied. In the CHO (Chinese hamster ovary) cell lines, it was observed that the glucan present in the GPI proteins induces oligomerization processes in the PS2 toxins but has a co-receptor function in the toxic action because it is also present in resistant cells to PS2Aa1 peptide [55]. Therefore, the APN is another very interesting receptor to investigate.

The h-APN receptor has been shown to have a high affinity to HCoV-229E. The HCoV-229E receptor-binding domain (RBD) binds at a site on h-APN called H-site. The HCoV-229E RBD binds to h-APN in its closed conformation, and structural comparison shows that the H-site does not differ between the open and closed conformations. It was also studied that there are three extended loops responsible for the binding of the receptor and the evolution of HCoV-229, and their close relatives are accompanied by changing loop-receptor interactions. Within loop 1, residues Cys^317^ and Cys^320^ form a disulfide bond that makes a stacking interaction with the side chains of residues Tyr^289^ and Glu^291^ in hAPN The most prominent of the remaining RBD–hAPN interactions are the salt bridge between residue Arg^359^ in loop 2 and residue Asp^315^ in hAPN and the interactions between residues Trp^404^ and Ser^407^ in loop 3 with residues Asp^315^ and Lys^292^ in hAPN; the importance of Trp^404^ in loop 3 as evidenced by the fact that mutating it also ablated binding [56].

The entry of the most active peptides into the cell may be mediated by interaction with receptors such as hAPN. Figure 5 shows that the colocalization of the peptide Loop1-PS2Aa peptide at 47.5 µM was in the nucleus of SW480 cells after 1 h of exposure, while the peptide P24-G274 was located in cytoplasmic regions in the same time of exposure, without affecting the nucleus. Likewise, results obtained at higher levels of cell adhesion demonstrated that the Loop1-PS2Aa peptide enters SW480 cells much faster than P24-G274 peptide in SW620 cells. Slater et al*.* [57] compared in an *in vitro* model of cancer progression, the SW480 and SW620 paired cell lines derived from a colon adenocarcinoma and its lymph node metastasis, respectively. Their chemosensitivity and the cancer stem cell (CSC) properties were investigated. The mentioned study determined that the SW480 and SW620 cells exhibited similar growth rates, although the SW480 cells were more migratory in wound healing assays on collagen and fibronectin matrices. SW480 and SW620 cells displayed similar CSC profiles, although, SW480 cells demonstrated significantly greater tumorsphere forming efficiency over SW620 cells. Tumorspheres derived from SW480 and SW620 cells also displayed differential sensitivity to 5-FU, oxaliplatin, geldanamycin, and novobiocin, which was not apparent when cells were grown under adherent conditions. These results suggest that although the two cell lines have similar levels of putative CSC populations, there are differences in their physiology that cannot be explained by these CSC levels.

Finally, according to the molecular docking simulations performed, an interaction is possible between the best experimental peptides, i.e., P264-G274 and Loop1-PS2Aa, and the h-APN receptor which is overexpressed in cancer cells. Even though the number of possible hydrogen bonds revealed the global simulations is low for the top residues, the situation changes significantly with the refinement models, where residues with more than 100 possible hydrogen bonds were found in 100 predicted models. Our results demonstrate that some regions of the domain I of PS2Aa1, represented in small peptide fragments of the protein, may be related to anticancer activity and initial adhesion with receptors. The latter results could be remarkable, in finding the main cell membrane receptor who interacts with the parasporins in human cancer cells. This strategy of protein fragmentation can give insights about the mechanism of action of PS2Aa1 and the role that each of its domains plays in the cytotoxic effect in different cancer cell lines. Furthermore, the present study constitutes a basis for the development of new analogs peptides or else conducting site-directed mutations of the PS2 protein to increase its anticancer potential. Additionally, Loop1-PS2Aa and P264-G274, peptides that exhibited stronger anticancer activity against SW480 and SW620 respectively and demonstrated high effectiveness and selectivity, are proposed as possible alternative as therapeutic agents for the treatment of colon cancer.

## Supplementary Material

Supplementary Figures S1-S5

## Data Availability

All data are available within the submitted manuscript.

## Abbreviations

5-FU: 5-fluorouracil
β-PFT: β-pore-forming toxin
ACP: anticancer peptide
CHO: Chinese hamster ovary
CRC: colorectal cancer
CSC: cancer stem cell
h-APN: human APN
PS2Aa1: Parasporin-2Aa1
