# Supplementary material for: Computational study, synthesis and evaluation of active peptides derived from Parasporin-2 and spike protein from Alphacoronavirus against colorectal cancer cells
Source: Biosci Rep. 2021 Dec 8;41(12):BSR20211964. doi: 10.1042/BSR20211964 (PMC8661510; doi:10.1042/BSR20211964)
Supplement: Supplementary Figures S1-S5 [file BSR-2021-1964_supp.pdf]

Supporting information

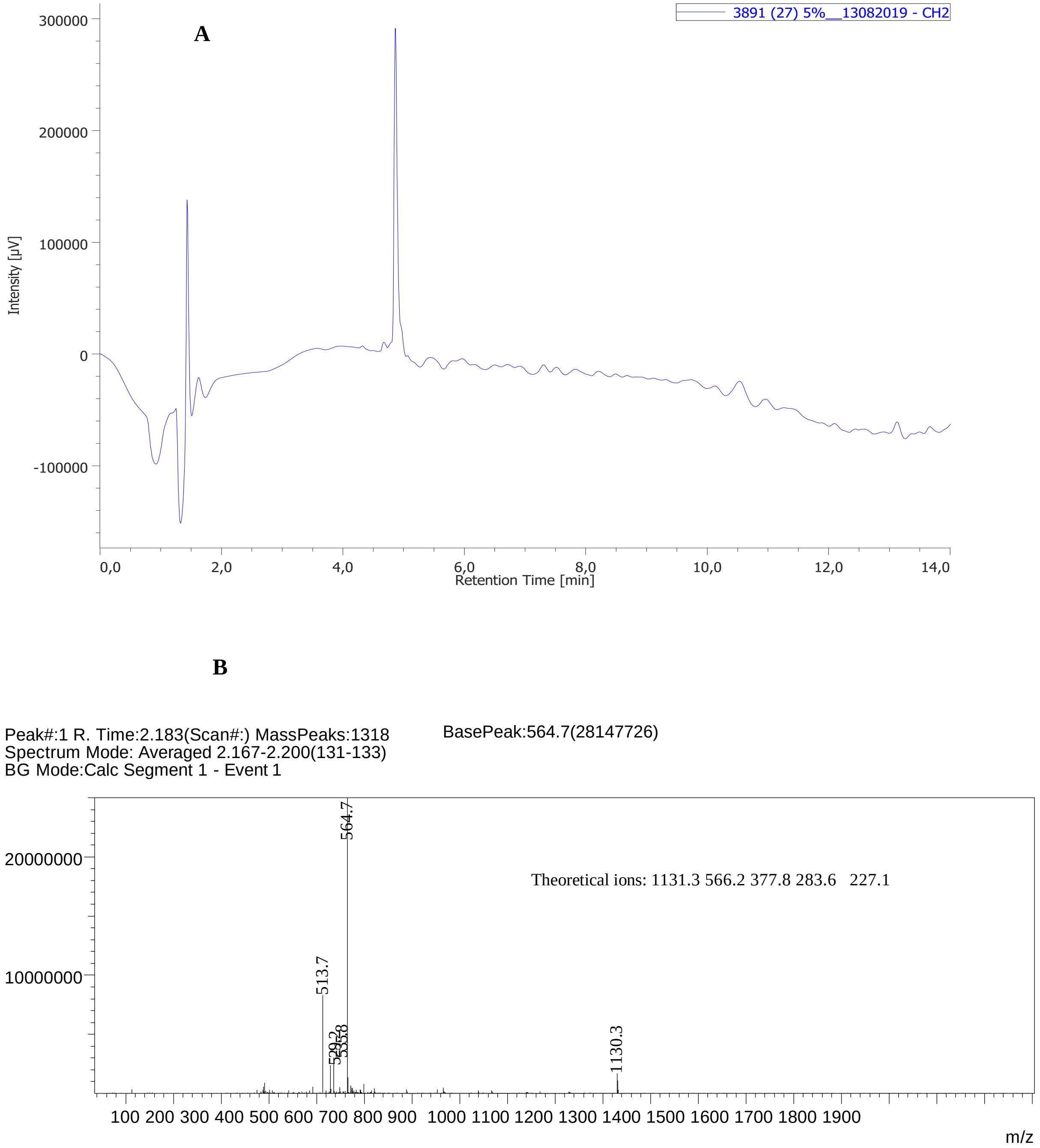

Figure S1. Characterization of P264-G274 peptide. A) Chromatogram spectra. B) mass spectra.

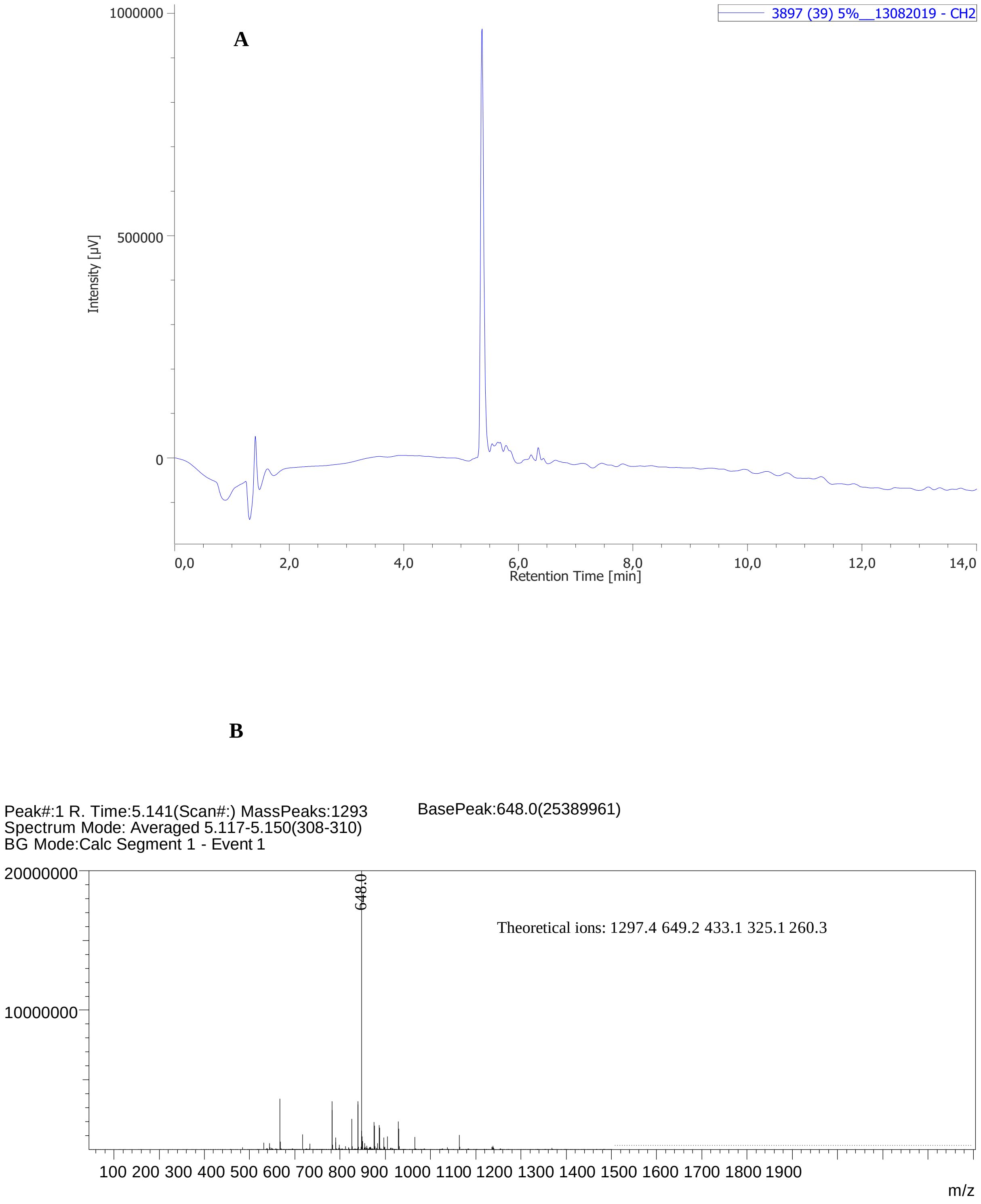

Figure S2. Characterization of Loop1-Ps2Aa peptide. A) Chromatogram spectra. B) mass spectra.

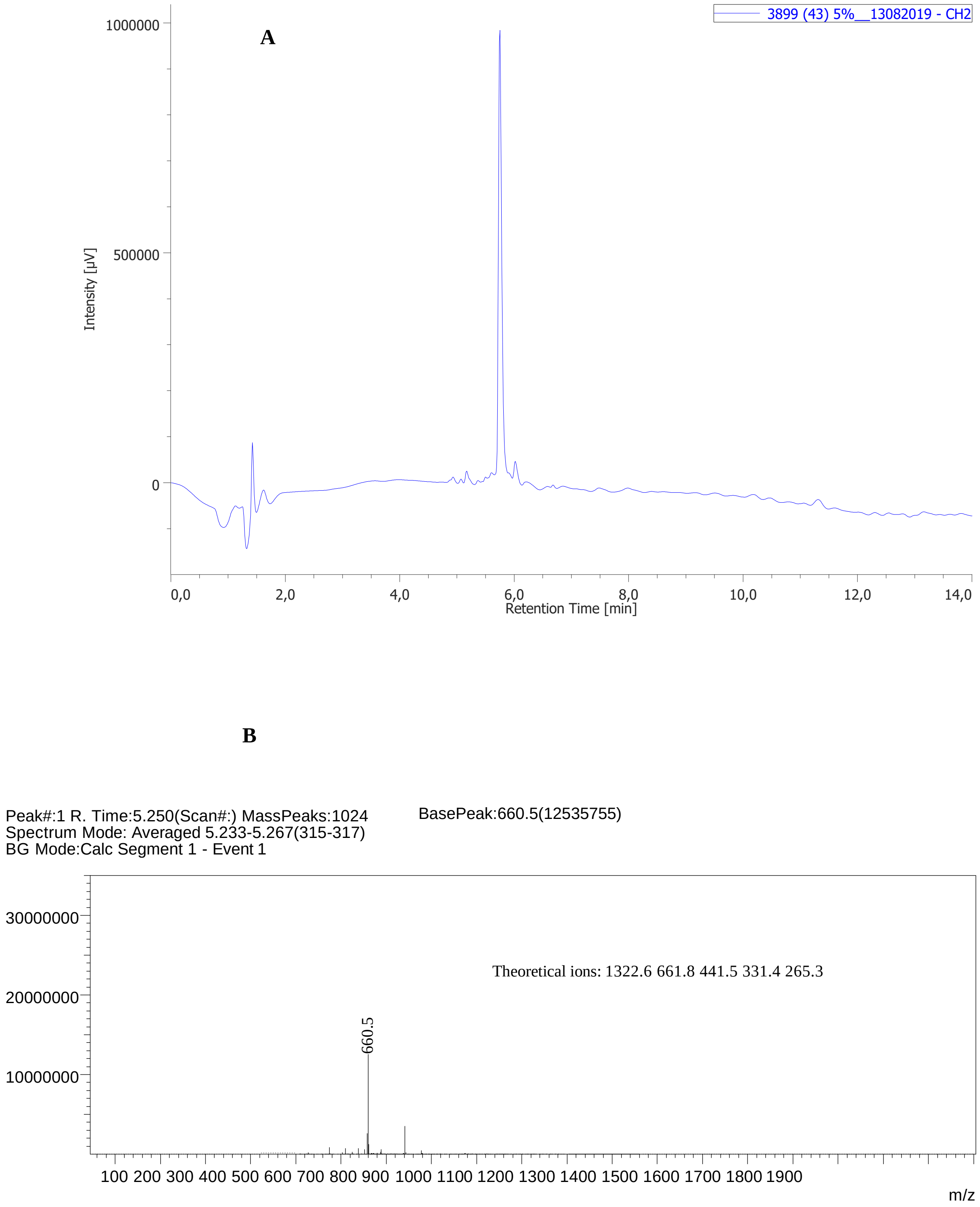

Figure S3. Characterization of Loop2-Ps2Aa peptide. A) Chromatogram spectra. B) mass spectra.

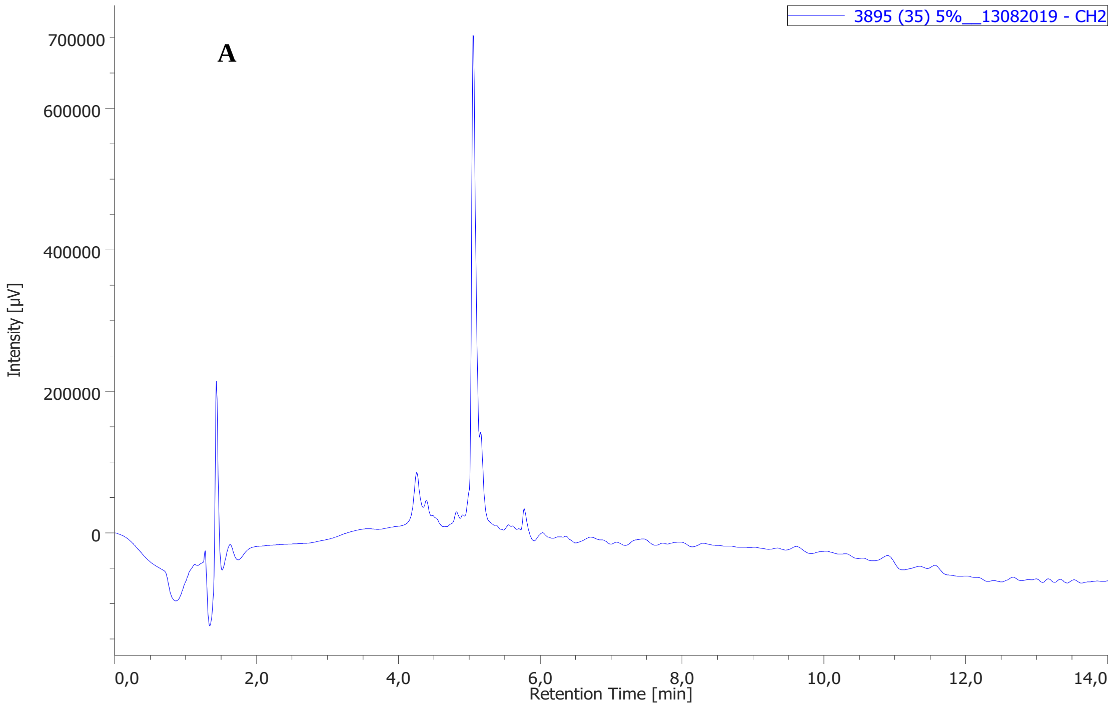

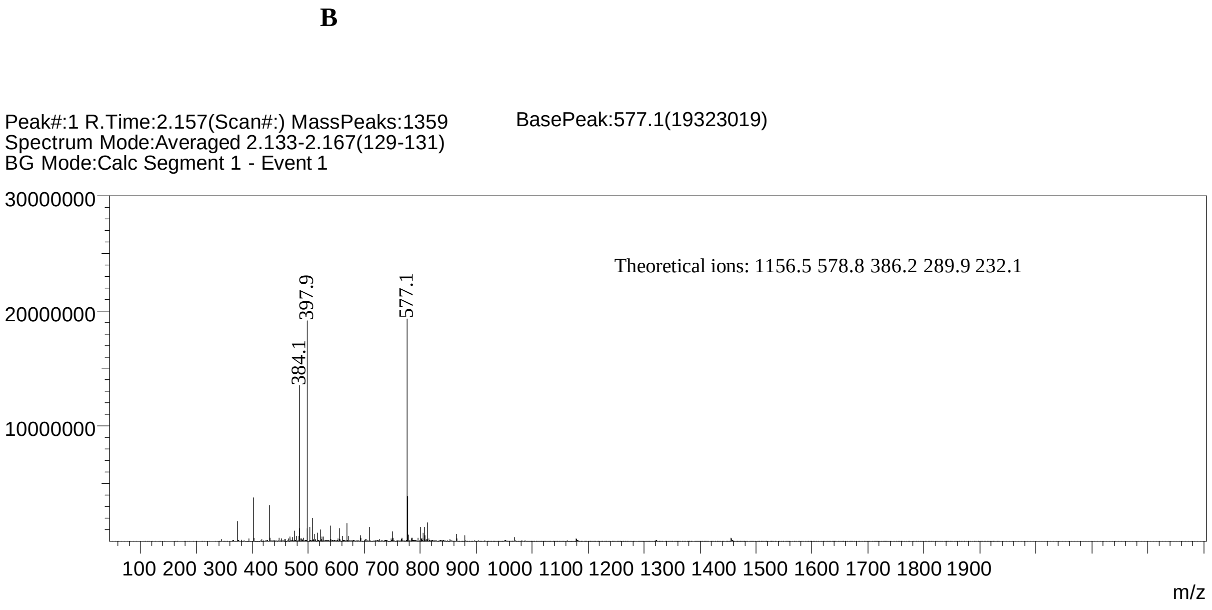

Figure S4. Characterization of Loop1-HCoV-229E peptide. A) Chromatogram spectra. B) mass spectra.

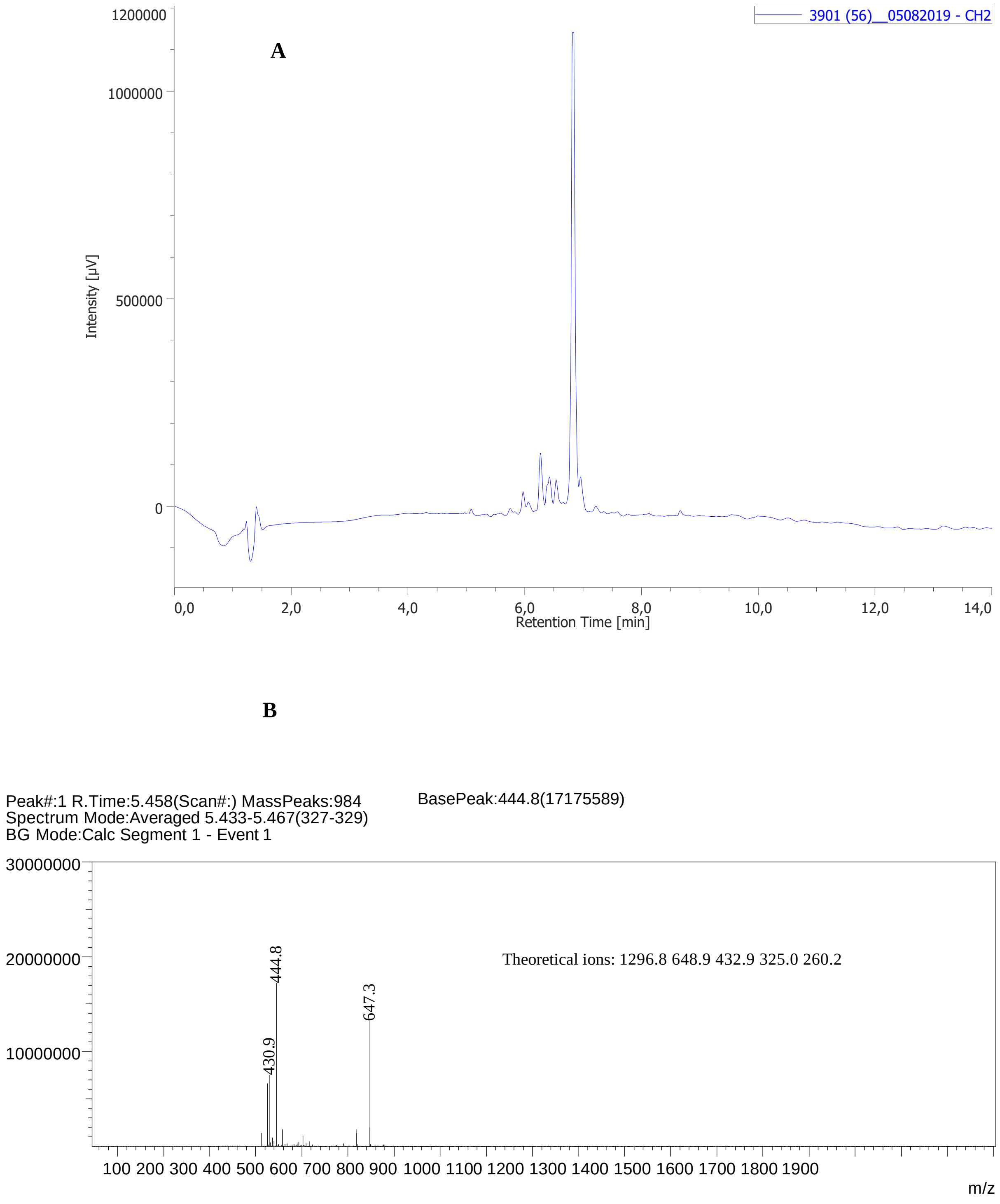

Figure S5. Characterization of A4W-GGN5 peptide. A) Chromatogram spectra. B) mass spectra.
